# Core patient-reported outcome measures for chronic pain patients treated with spinal cord stimulation or dorsal root ganglia stimulation

**DOI:** 10.1186/s12955-023-02158-2

**Published:** 2023-07-20

**Authors:** Frank Huygen, Jonathan M. Hagedorn, Steven Falowski, David Schultz, Jan Vesper, Robert D. Heros, Denis G. Patterson, Soroush Dehghan, Erika Ross, Anahita Kyani, Misagh B. Mansouri, Jan Willem Kallewaard

**Affiliations:** 1 Erasmus University Medical Center, Dr. Molewaterplein 40, 3015 GD Rotterdam, Postbus 2040, 3000 Rotterdam, CA Netherlands; 2 Department of Anesthesiology and Perioperative Medicine, Mayo Clinic, Rochester, MN USA; 3 Neurosurgical Associates of Lancaster, Lancaster, PA USA; 4 Nura Pain Clinic, Minneapolis, MN USA; 5 Heinrich-Heine-Universität Düsseldorf, Düsseldorf, Germany; 6 Spinal Diagnostics, Tualatin, OR USA; 7 Nevada Advanced Pain, Reno, NV USA; 8 Abbott Neuromodulation, Austin, TX USA; 9 Rijnstate Hospital, Arnhem, Netherlands

**Keywords:** Chronic pain therapy, Spinal cord stimulation, Patient-reported outcomes, Digital health, Confirmatory factor analysis, Dimensionality reduction, Questionnaire burden

## Abstract

**Background:**

Neurostimulation is a highly effective therapy for the treatment of chronic Intractable pain, however, due to the complexity of pain, measuring a subject’s long-term response to the therapy remains difficult. Frequent measurement of patient-reported outcomes (PROs) to reflect multiple aspects of subjects’ pain is a crucial step in determining therapy outcomes. However, collecting full-length PROs is burdensome for both patients and clinicians. The objective of this work is to identify the reduced set of questions from multiple validated PROs that can accurately characterize chronic pain patients’ responses to neurostimulation therapies.

**Methods:**

Validated PROs were used to capture pain, physical function and disability, as well as psychometric, satisfaction, and global health metrics. PROs were collected from 509 patients implanted with Spinal Cord Stimulation (SCS) or Dorsal Root Ganglia (DRG) neurostimulators enrolled in the prospective, international, post-market REALITY study (NCT03876054, Registration Date: March 15, 2019). A combination of linear regression, Pearson’s correlation, and factor analysis were used to eliminate highly correlated questions and find the minimal meaningful set of questions within the predefined domains of each scale.

**Results:**

The shortened versions of the questionnaires presented almost identical accuracy for classifying the therapy outcomes as compared to the validated full-length versions. In addition, principal component analysis was performed on all the PROs and showed a robust clustering of pain intensity, psychological factors, physical function, and sleep across multiple PROs. A selected set of questions captured from multiple PROs can provide adequate information for measuring neurostimulation therapy outcomes.

**Conclusions:**

PROs are important subjective measures to evaluate the physiological and psychological aspects of pain. However, these measures are cumbersome to collect. These shorter and more targeted PROs could result in better patient engagement, and enhanced and more frequent data collection processes for digital health platforms that minimize patient burden while increasing therapeutic benefits for chronic pain patients.

**Supplementary Information:**

The online version contains supplementary material available at 10.1186/s12955-023-02158-2.

## Introduction

The individual and social burdens associated with chronic pain have been escalating globally. Chronic pain affects approximately one in five adults worldwide and estimates of chronic pain among U.S. adults range from 11 to 40% [1, 2]. In addition to unpleasant sensory experiences, many subjects often suffer emotional and psychological distress due to the interference of pain with activities of daily life and with sleep [3, 4]. Psychological distress refers to a unique experience of discomfort and unpleasant feelings that can manifest in various ways such as sadness, anxiety, distraction, self-criticism, and in the most extreme cases- psychotic symptoms [5]. Psychological distress in chronic pain is believed to be related to stressor factors such as the ability of persistent chronic pain to stimulate the body through mediating mechanisms such as sleep quality [6] and self-perception of health [7, 8]. Accurate pain measurement facilitates early diagnosis, disease progression monitoring, and therapeutic efficacy evaluation; thus, it is key to the management of chronic pain.

The intensity of pain is usually evaluated in clinical settings by subjective unidimensional scales such as the Numeric Rating Scale (NRS) and the Visual Analog Scale (VAS). These pain scales can be useful in assessing a person’s acute or sudden pain intensity. However, these tools can sometimes oversimplify the pain assessment process. Nonetheless, chronic pain often affects other dimensions of a patient’s life such as sleep, physical function, psychological health, and quality of life. Accurate pain measurement facilitates early diagnosis, disease progression monitoring, and therapeutic efficacy evaluation; thus, it is key to the management of chronic pain. Appropriately selected patient-reported outcomes (PROs) are effective tools to measure treatment efficacy using patients’ pain intensity, psychological metrics, disability and physical function metrics, and health-related quality of life scores [9]. Validated scales such as the Patient-Reported Outcomes Measurement Information System (PROMIS), a comprehensive set of physical, mental, and global health metrics, have been developed to provide a comprehensive and multidimensional measurement of pain. As recommended by the IMMPACT group, these metrics are becoming standard tools to measure pain in neurostimulation clinical trials, complementing unidimensional metrics such as NRS and VAS.

Neurostimulation therapy, in this paper referred to as both Spinal Cord Stimulation (SCS) or Dorsal Root Ganglia (DRG) stimulation, has been very effective in treating chronic pain [10–12]. Recent advancements in SCS, including different waveforms [13, 14], have increased the overall effectiveness of SCS as a treatment modality for chronic pain and psychological disorders [15, 16]. Despite recent advances, measuring subjects’ long-term response to neurostimulation therapy in a realistic and impactful manner remains difficult [17]. Prior research has emphasized the need for improved metrics to better characterize the long-term patient response to neurostimulation therapy and overall subject satisfaction with the therapy through a combination of global health measures and composite health scores [18–21]. In addition, more frequently collecting PROs via targeted short questions could enhance symptom monitoring to improve long-term therapy success by timely notifying patients and their managing clinicians of any changes in response to neurostimulation therapy.

In the digital health era, easily obtaining frequent information updates regarding a patient’s health condition, with minimal patient and clinician effort, is of paramount importance. Collecting multiple PROs to accurately measure the impact of pain on different aspects of patient’s lives can be burdensome for patients, research staff, and clinicians. The lengthy list of questions is time-consuming and subsequently reduces the likelihood that all the questions will be appropriately and accurately answered. This manuscript aims to identify a subset of questions selected from the commonly collected PROs for chronic pain that can be used to accurately model and characterize a patient’s response to neurostimulation therapies.

## Methods

Data for our analysis were extracted from the ongoing prospective, multicenter, international REALITY (Long-Term Real-World Outcomes Study on Patients Implanted with a Neurostimulator) study (NCT03876054). Before initiating the study, an Institutional Review Board or Ethics Committee approval was received at each site and all patients were provided with written informed consent. The devices used in this study are FDA-approved or approved by a corresponding national agency for this indication. Study enrollment is still ongoing, and subjects will be followed for five years from the time of permanent implant. Study visits occur at enrollment, at baseline, peri-operatively, and at six months, one year, and yearly thereafter until the subject has been followed for five years post-implantation.

The eligibility criteria included a baseline pain score ≥ 6 on the 0–10 Numerical Rating Scale (NRS) and scheduled for implantation of an Abbott SCS or DRG neurostimulation system (Abbott Neuromodulation, Austin, Texas) within 60 days of the baseline visit. The study inclusion criteria for REALITY were designed with few restrictions on the pain indication as allowed by the regulatory bodies in each geographical region and according to standard clinical practice to replicate the range of complex patients that would be seen in everyday medical practice.

Demographics, chronic pain history, and pain etiologies were collected at baseline. Various patient-reported outcome measures, as described in detail below, were collected at baseline and each follow-up study visit per the IMMPACT recommendations [22] to capture the effects of therapy on subjects’ pain, function, disability and mental health. Pain intensity was measured using NRS, where 0 is no pain and 10 is the worst pain imaginable [23]. Physical function and disability were measured with the 10-item Oswestry Disability Index (ODI) [24]. Each section in the scale covers a different domain (pain intensity, personal care, lifting, walking, sitting, standing, sleeping, sex life, social life, and traveling). Emotional distress was assessed with the 13-item Pain Catastrophizing Scale (PCS) that yields a total score and three domains assessing rumination, magnification, and helplessness [25]. PROMIS-29 was used to assess the following nine domains: Physical Function and Ability to Participate in Social Roles and Activities, as well as the seven days average of subject’s Depression, Anxiety, Fatigue, Sleep Disturbance, Pain Interference, and Pain Intensity [26, 27]. Patient Global Impression of Change (PGIC) [28], a 7-question scale to assess patient global health and subject satisfaction with pain relief from the neurostimulation device was also collected at each follow-up. All PROs were entered electronically through the Abbott study portal by the research coordinator. The clinical study team routinely monitored study site compliance for protocol deviation trends. In cases where protocol compliance issues were identified at individual sites during this routine monitoring, the study team provided protocol re-training and conducted on-site visits to review the data collection quality with the site staff.

We implemented the principal component analysis (PCA) dimensionality reduction as an exploratory way to identify similarities across multiple PROs. PCA is a statistical method used for large datasets to reduce the dimensionality of the data while increasing interpretability with minimal data loss. Survey questions from all scales were treated as unique entries. Data points from various scales were standardized using the z-score (standard score) prior to this analysis. The z-score describes the fractional distance between a data point and the population means in terms of standard deviation units. We performed linear regression analysis to assess the relationship across multiple PROs such as ODI and PROMIS-29 where PCA identified clusters with common questions.

To further examine the questions selected by the PCA within each PRO, we ran Pearson’s correlation [29] to select the highly associated questions within the PCS and PROMIS-29 questionnaires. The correlation analysis was used to measure the strength and direction of linear relationships between pairs of survey questions in long-form versions of both PROMIS-29 (29 questions and nine domains) and PCS (13 questions and three domains) within the scale domains as a constraint. The items for the short scales were selected based on eliminating questions in each domain with an inter-item correlation greater than 0.65. We used an absolute correlation value of 0.65 within each domain of the scales for both PPCS and PROMIS-29 to identify highly correlated questions [30]. Any question item within a domain with lower than this correlation threshold was kept unchanged to ensure only the highly correlated items were eliminated. Next, we used Confirmatory Factor Analysis (CFA) to verify the factor structure of each PRO’s questions in their defined domains [31]. The chi-squared test is used to test the relationship between expected and observed covariance matrices. The model fit for CFA was evaluated using the Root Mean Square Error Approximation (RMSEA, acceptable fit if < 0.06, marginal fit if > 0.08 and < 0.1, poor fit > 0.1), the Comparative Fit Index (CFI, acceptable fit if > 0.95, poor fit if < 0.90, otherwise marginal) and the Tucker Lewis Index (TLI, acceptable fit if > 0.95, poor fit if < 0.90, otherwise marginal) [32].

**Fig. 1:**
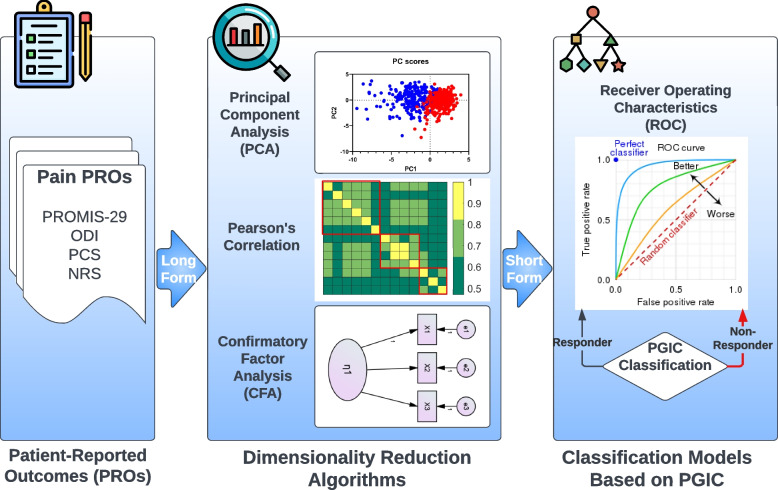
Analysis and validation flowchart. Statistical analyses and dimensionality reduction algorithms were performed to select the short-form questions. The left panel shows the long-form validated patient-reported outcomes (PROs) as inputs. The middle panel shows different algorithms used to generate the short-form questions. The right panel shows the ROC classifier used to differentiate model performance for both long-form and selected short-form questions

Lastly, we evaluated the patient’s response to neurostimulation therapy using both the original long-form and selected short-from questions using Receiver Operating Characteristics (ROC) analysis on the changes of all PRO values at each follow-up visit from baseline. Subject response to neurostimulation therapy was evaluated based on PGIC categories. Subjects who selected “No change (or the condition has gotten worse)”, “Almost the same, or hardly any change at all”, “A little better, but no noticeable change”, and “Somewhat better, but the change has not made a real difference” were considered non-responders. Subjects who selected “Moderately better, and a slight but noticeable change”, “Better and a definite improvement that has made a real and worthwhile difference”, and “A great deal better and a considerable improvement that has made all the difference” were considered responders. The classification accuracies between the original long-form and selected short-form questions were then compared using the Area Under the Curve (AUC) from the ROC analysis. Figure 1 illustrates the flow diagram of the statistical analyses performed to select and validate the short-form questions.

## Results

At the time of data collection, 538 patients at 53 investigational sites (28 in the United States, 23 sites in the European Union, and two sites in Australia) had completed at least one follow-up visit. Of those, 391 subjects were implanted with an SCS, and 118 subjects were implanted with a DRG neurostimulator system. Baseline characteristics and pain etiologies for all subjects are summarized in Table 1. A total of 872 follow-up visits were included in the analysis in this paper. As of the data cut-off for this study, the follow-up visits included 452 subjects at 6 months, 305 at 1 year, and 115 visits at 2 years. Each study visit was treated as a separate data point in the analysis. A summary of all collected PROs (NRS, ODI, PCS, and PROMIS-29) and a comparison of the mean of each PRO at all study visits and their corresponding significant differences (marked for all pairs with *p*-values < 0.001) is presented as a Supplementary Table S1.

**Table 1:** Baseline characteristics and chronic pain etiologies of subjects enrolled in the REALITY study

**Baseline Characteristics**		**N (count) or mean (± SD)**
Sex at Birth	Male	197
	Female	312
Age at time of consent	Years	59.75 ± 14.06
Weight	Kg	87.99 + 22.48
Height	cm	168.65 + 10.14
Chronic Pain History	Years	10.67 ± 11.40
Pain Etiology^a^	Back/lower Limb PSPS^a^ type I	74
	Back/lower Limb PSPS^a^ type II	192
	Radiculopathy	53
	CRPS^b^- I	66
	CRPS^b^- II (Causalgia)	40
	Chronic Post-surgical Pain	11
	Neck/Upper Limb PSPS^a^ type I	6
	Neck/Upper Limb PSPS^a^ type II	7
	Peripheral Neuropathy^c^	31
	Other Chronic Pain^d^	29
Work Status	Disabled	76
	Not Working	301
	Part-time	40
	Full-time	92

^a^*PSPS* Persistent Spinal Pain Syndrome, *Type I* Non-surgical, *Type II* Surgical

^b^*CRPS* Complex Regional Pain Syndrome

^c^(Poly)neuropathies including painful diabetic polyneuropathy and post herpetic neuralgia

^d^Other Chronic Pain” included Angina, Critical Limb Ischemia, Visceral pain, and post-amputation pain

**Fig. 2:**
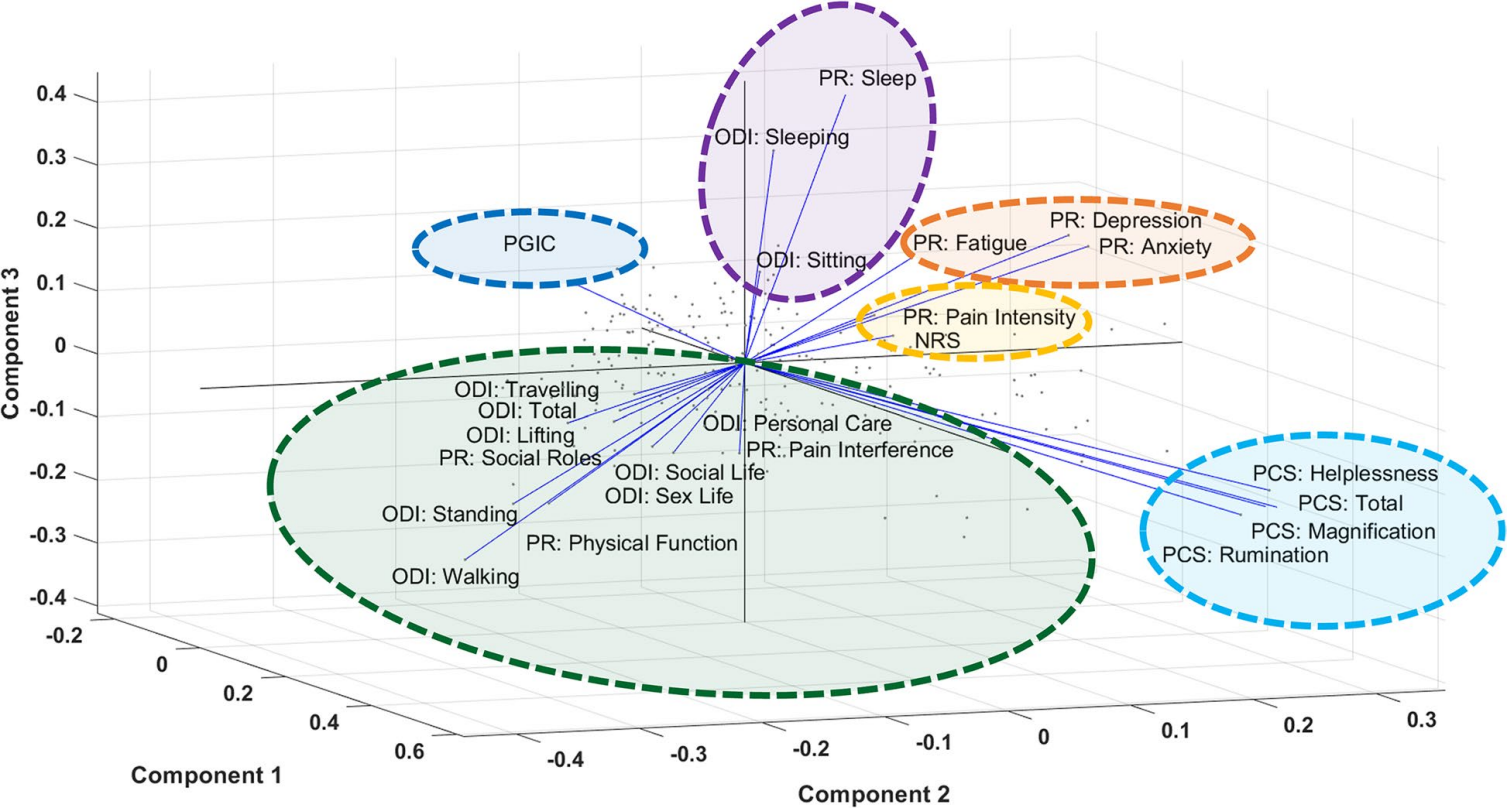
The PCA loading plot of the first 3 principal components. PCS, PROMIS29, PGIC, and ODI scales and their sub-domains were selected for this analysis. PR is an abbreviated version of the PROMIS-29; Total stands for the total score for each scale of ODI and PCS. The black dashed line shows the manual grouping of the domains based on the loading weights in all three dimensions

The PCA analysis on all domains of PROMIS 29, PCS, ODI, NRS, and PGIC revealed clustering of meaningful questions. The top five principal components explained 85% of the data: 55.4%, 10.8%, 7.8%, 6.1%, 4.4%, and 3.9% for each principal component, respectively. As a measure of domain proximity, the 3-dimensional PCA loading plot shown in Fig. 2 for the first three principal dimensions identified several meaningful clusters (highlighted with the dashed black line, Fig. 2): emotional components such as PCS total score and the subcategories of Rumination, Magnification, and Helplessness; Physical activities and disabilities components such as PROMIS-29 Social Roles, Pain Interference, and Physical Function, ODI Personal Care, Social Life, Sex Life, Traveling, Lifting; PROMIS-29 Anxiety, Depression, and Fatigue; PROMIS-29 Sleep disturbance with ODI Sleep and Sitting; and NRS and PROMIS-29 pain intensity. Two-dimensional (2D) projections of the top five principal components loading plots used to cluster domains of different scales are included in Supplementary, Figure S1.

As also highlighted in the largest cluster in Fig. 2, the majority of ODI domains and ODI total score clustered closely with the PROMIS-29 Pain Interference, Social Roles, and Physical Function domains. The PCA clustering was also confirmed by the correlation results between PROMIS-29 and ODI scales. Regression analysis showed high correlations between ODI and PROMIS-29 Pain Interference (*R* = 0.78), Social Roles (*R* = -0.72), and Physical Function (*R* = -0.73). Supplementary, Figure S2, shows the regression analysis of the ODI total score versus the PROMIS-29 domains of Physical Function, Social Roles, and Pain Interference. Each blue dot shows the variability of individual subjects for these two scales.

**Fig. 3:**
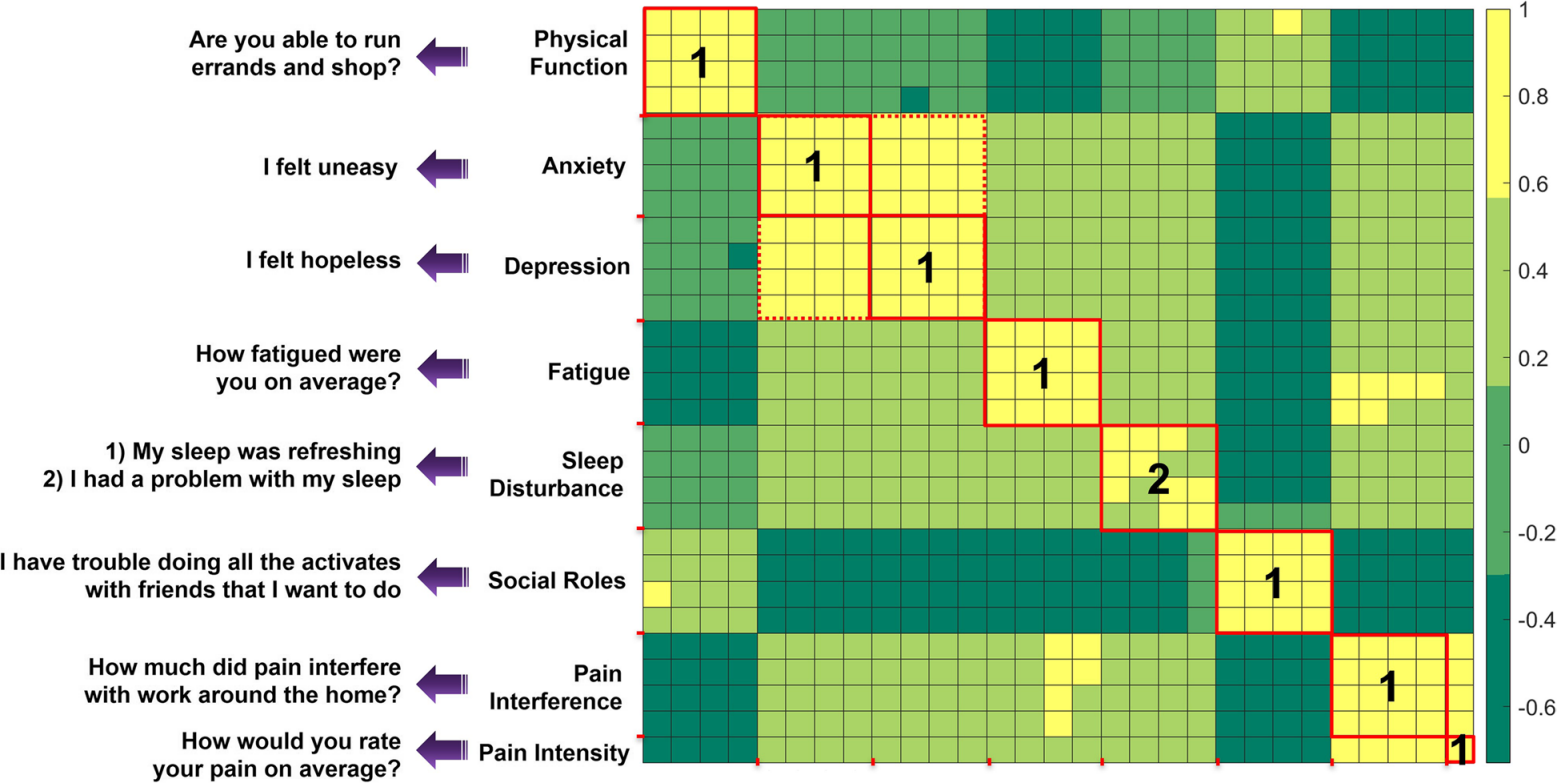
Correlation heatmap for PROMIS-29 domains. The colors are based on the correlation thresholds, yellow (strong positive correlation), light green (weak to moderate positive correlation), and dark green (strong negative correlation). The red solid line borders separate the PROMIS-29 domains to highlight the correlations within each PROMIS domain. The red dashed line shows the borders for the strongly correlated combined PROMIS-29’s Anxiety and Depression domains

**Fig. 4:**
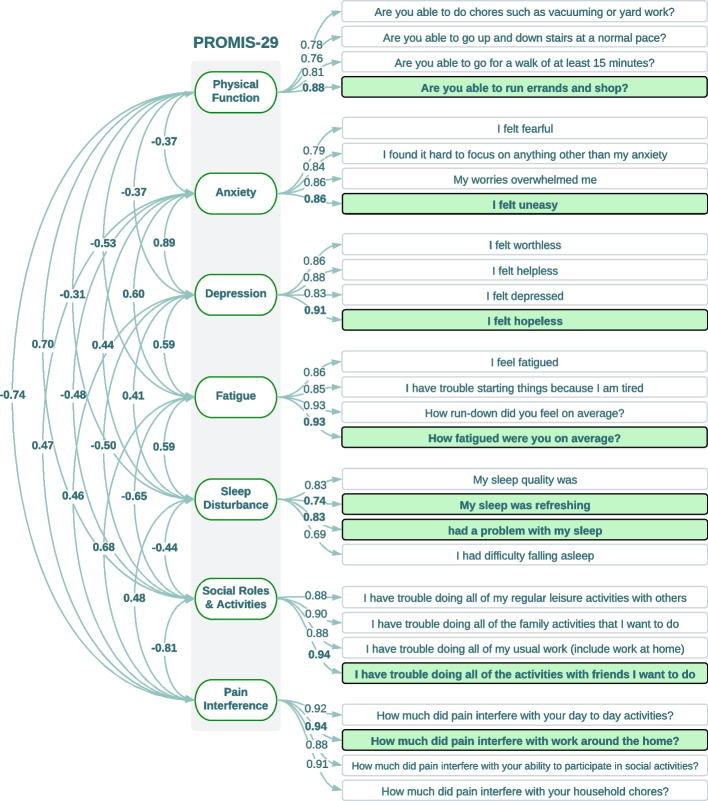
CFA flowchart for each domain of PROMIS-29. Short-form questions were selected based on the highest standardized factor loadings in each domain

**Fig. 5:**
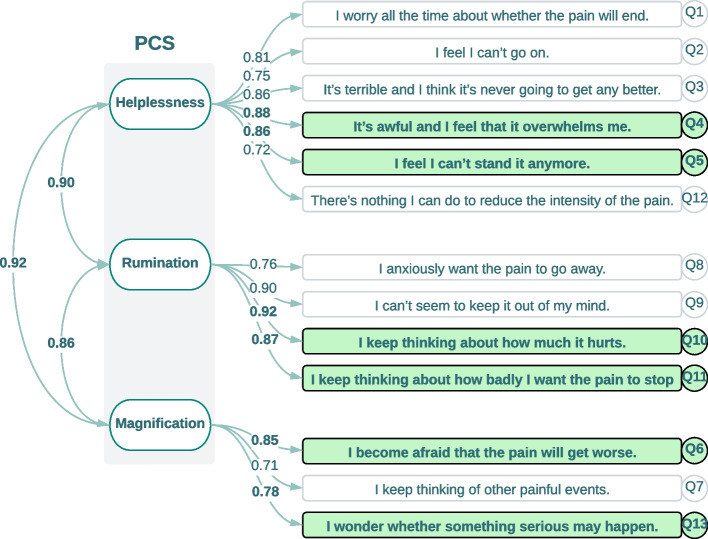
CFA flowchart for each domain of the PCS. Short-form questions were selected based on the highest standardized factor loadings in each domain

Pearson’s correlation analysis exposed several highly correlated clusters (R ≥ 0.65) within each domain of PROMIS-29 and PCS. Each PROMIS-29 domain includes four questions. A single question within each domain was selected apart from the sleep domain, where two questions were selected (Fig. 3). The pain intensity was also selected as a separate question. All original 29 questions and the nine questions selected from eight domains from the short form are shown in Fig. 4. Depression and anxiety questions of PROMIS-29 are highly correlated. Physical function and social roles showed a strong negative correlation with the rest of the PROMIS-29 domains. Factor analysis using CFA also returned identical selections for the nine questions in the PROMIS-29 short form. The standardized factor loadings show how well each question is represented by the observed variables. Additionally, CFA analysis provided weights across eight domains of PROMIS-29 (Fig. 4). Similarly, for PCS, within each domain of rumination, magnification, and helplessness, two questions were selected. The items for the short scales were selected based on the highest corrected inter-item correlation value. We included the least correlated question in the magnification category to preserve as much information as possible. CFA also returned six questions from the PCS short form (Fig. 5). The parameters for the model fit for PROMIS-29 (RMSEA = 0.054 [0.051–0.58], CFI = 0.963, TLI = 0.954), and PCS (RMSEA = 0.085 [0.077–0.092], CFI = 0.960, TLI = 0.950) met the evaluation criteria.

The ROC analyses showed almost identical classification accuracy for modeling patient response to neurostimulation therapy on the PGIC scale using both long and short versions of PROMIS-29 and PCS scales. The classification accuracy was quantified with the area under the curve (AUC) for both questionnaires. Table 2 summarizes the AUC and 95% confidence interval (CI) lower and upper bound for different domains of PROMIS-29 and PCS.

**Table 2:** Classification model performance comparison. Area under the curve (AUC) was used to compare short- and long-form of the PROMIS-29 and PCS derived from the ROC classification model

	**ROC**					
	**(Long-Form)**			**(Short-Form)**		
**Test Result Variable (s)**	AUC	95% CI		AUC	95% CI	
		Lower Bound	Upper Bound		Lower Bound	Upper Bound
NRS	0.744	0.701	0.787	0.744	0.701	0.787
PROMIS-Physical Function	0.699	0.656	0.743	0.665	0.619	0.711
PROMIS-Anxiety	0.596	0.546	0.645	0.559	0.509	0.608
PROMIS-Depression	0.556	0.505	0.607	0.572	0.52	0.623
PROMIS-Fatigue	0.63	0.582	0.678	0.592	0.543	0.641
PROMIS-Sleep Disturbance	0.593	0.544	0.642	0.593	0.543	0.642
PROMIS-Social Roles	0.693	0.648	0.738	0.672	0.625	0.718
PROMIS-Pain Interference	0.753	0.711	0.794	0.728	0.684	0.771
PROMIS-Pain Intensity	0.75	0.707	0.792	0.75	0.707	0.792
PCS Total Score	0.701	0.656	0.746	0.704	0.659	0.75
PCS-Helplessness	0.695	0.65	0.74	0.708	0.663	0.752
PCS-Rumination	0.692	0.646	0.737	0.658	0.61	0.707
PCS-Magnification	0.641	0.592	0.691	0.649	0.6	0.697

## Discussion

In this work, we used statistical models and dimensionality reduction techniques, such as CFA, PCA, and Pearson’s correlation to identify the reduced set of questions from the validated PROs to model subjects’ response to neurostimulation therapy. Our results suggest that selected questions from multi-dimensional PROs that cover different aspects of pain combined into a short-form survey questions are sufficiently good at quantifying overall meaningful improvement in chronic pain populations as the long-form versions of the surveys. There are many highly correlated questions within different domains of PROs such as PCS, PROMIS, and ODI. Although these questions are important factors to provide comprehensive details on subjects’ functioning, psychological health, and pain, they do not necessarily provide additional information on how well a subject is responding to neurostimulation therapy. Our analysis shows the shorter version of the questions proposed here has just as much predictive power as the original longer versions for classifying patient response to neurostimulation therapy using the PGIC scale. In addition, in consideration of the IMMPACT clinical guidelines for the measurement of pain, we kept all the domains of the PROMIS-29 and PCS scales and selected the questions within each domain to make sure we were not neglecting crucial information due to the nature of our data set. Although there are several validated questionnaires for characterizing patients’ emotional distress, PCS was used as the main surrogate for measuring emotional distress. This was due to previous studies that showed pain catastrophizing is the most consistent psychosocial factor predictor of chronic pain and a higher level of catastrophizing is often associated with the intensity of chronic pain intensity and disability [33].

The short form comprising questions selected from the PROMIS-29 and PCS proposed in this study have many overlaps with the already validated shorter versions of PCS (PCS-3, PCS-4, and PCS-6) [34, 35] and PROMIS (PROMIS-10) [36]. PROMIS-10 is a global health metric for assessing healthcare-related quality of life for the general population. One of the main differences between the nine questions selected from our data set to comprise our short form and the PROMIS-10 is the lack of a sleep category in PROMIS-10 [37]. The interrelationship between sleep disturbance and chronic pain is well established [38, 39]. Additionally, our algorithm selected two sleep questions due to a lower correlation among the four original sleep domains in PROMIS-29. Similarly for the PCS, two questions from each domain of helplessness, magnification, and rumination were selected. These questions include all three questions of the validated PCS-3 but only three questions in PCS-6, and two questions in PCS-4. Our results suggest that the number of questions in the longer versions of the PROs could be redundant and thus could be pared down by ultimately consolidating questions from multiple, validated, long-form PROs into one short form that may be equally accurate in terms of quantifying subject response to neurostimulation therapy as compared to the use of the full questionnaires. It may also be surmised that a short form derived from our analysis would be more suited than would be a conglomerate of questions selected from the previously validated short versions of PROs already reported in the literature.

Our principal component analysis (PCA) suggests that there is overlap across questions obtained from multiple PROs that can be reduced to optimize the process. PCA results showed a clear representation of different aspects of chronic pain such as physical function and disability, psychological affects, sleep and fatigue, and pain intensity that can be captured using different overlapping questions from multiple PROs. The ODI score and its sub-questions are strongly correlated with multiple domains of the PROMIS-29. Prior research established similar correlations between PROMIS physical function and ODI in patients with back or neck pain [40] as well as a strong correlation with PROMIS pain interference and a moderate correlation with depression [41]. Similarly, our data support a strong negative correlation with physical function (*R* = -0.73; *P* < 0.001) and social roles (*R* = -0.72; *P* < 0.001), and a strong positive correlation with pain interference (*R* = 0.78; *P* < 0.001). These findings strongly suggest the possibility of characterizing subjects’ physical function and disability measures in response to neurostimulation therapy without collecting the ODI. In addition, the choice of the PROs we used in this work was from our post-market REALITY study. These PROs were selected to capture different aspects of pain for patients with very broad pain etiologies in as close to real-world scenarios as possible. The study results suggest, even with a broad selection of chronic pain patients included in our analysis, a limited set of questions captured from multiple PROs can provide adequate information for measuring therapy success.

Our study has limitations. The data used for training and testing the classifications of PGIC were both from a post-market study and was limited to a single intervention. Despite this, the study included patients from 53 international sites and the study was designed with very few exclusion criteria for patient selection in order to replicate the range of complex patients that would be seen in everyday clinical practice. This makes our dataset more heterogenous and less prone to common overfitting issues. Additionally, the conclusions made here have not been tested in all neuromodulation designs or other commercially available programming waveforms; the majority of the subjects in this study were programmed with paresthesia-free DeRidder Burst [16] with the SCS devices or sub-threshold tonic [42] for the DRG devices as their primary neurostimulator waveform. In this paper, we utilized statistical approaches based on psychometric analyses such as CFA, PCA, and Pearson’s correlation to evaluate the clinical utility and sensitivity of patient-reported outcome measures for the assessment of different aspects of pain in patients treated with spinal cord stimulation. Future studies to evaluate the clinical properties of these assessment instruments using clinimetric principles for the development and validation process of patient-reported outcome measures might be needed [43, 44]. In addition, future testing and validating of our approach on large independent data sets can increase the generalizability and robustness of our methodology.

PROs are important subject measures to evaluate physiological and psychological aspects of pain, but these measures are cumbersome to collect and are sometimes redundant. Reducing the number of questions collected through the surveys likely would increase patient engagement and could help create patient-centric digital health products. Furthermore, the validated long-form and short-form PROs measuring subjective data are limited in distinctiveness. Current subjective measures (using both short and long versions of PROs) have limitations in quantifying subject responses to therapy. Different variables in both models showed AUC around (0.55 to 0.75), demonstrating moderate classification power. Future work will be focused on integrating objective measures gleaned from wearable technologies with subjective data derived from the short form of PROs. Adding objective measurements could improve the accuracy of classification models and enable us to move toward a more personalized therapy with a limited burden on both patients and clinicians.

## Conclusions

PROs are important subjective measures to evaluate the physiological and psychological aspects of pain. However, these measures are cumbersome to collect. The reduced number of questions selected using our mathematical algorithms demonstrated almost identical accuracy for predicting chronic pain outcomes when compared with using longer validated questionnaires. The shorter and more targeted PROs could potentially result in better patient engagement, enhanced data collection processes, and ultimately increase patient satisfaction with neurostimulation therapy. Testing our approach on independent and more heterogenous data sets and combining meaningful outcomes with objective measures will be the next step in validating our approach and moving away from solely relying on the more subjective NRS/VAS score. This work highlights the potential to move toward a digital health platform that minimizes patient burden while increasing therapeutic benefits for chronic pain patients.

## Trial registration

Data for our analysis were extracted from the ongoing the prospective, multicenter, international, post-market REALITY (Long-Term Real-World Outcomes Study on Patients Implanted with a Neurostimulator) study (Trial Registration Number: NCT03876054, Trail Registration Date: March 15, 2019).

## Supplementary Information

**Additional file 1: Table Supplementary 1.** Summary of all collected PROs (NRS, ODI, PCS, and PROMIS-29) and comparison of the mean of each PRO at all study visits and their corresponding significant differences.

**Additional file 2: Figure Supplementary 1.** Two-dimensional (2D) projection of the top 5 principal components used to cluster the questionnaires. Only ODI total score is included in the 2D projection of the principal components.

**Additional file 3: Figure Supplementary 2.** Regression analysis of the ODI total score versus the PROMIS-29 domains of Physical Function, Social Roles, and Pain Interference. Each blue dot shows an individual subject data point for these two scales. The box and whisker plots show the variation of both scales; the minimum, the maximum, the first and third quartiles, and the median are illustrated with a solid black line.

## Data Availability

The REALITY study is still ongoing. The data will be available upon reasonable request or by the completion of the study according to the study protocol if a written request is made to and granted in writing by Abbott at Abbott’s sole discretion. The requestor should include their name, title, contact information, and the institution they work for as well as the specifics regarding the use and necessity of the requested dataset.
