# Supplementary material for: Core patient-reported outcome measures for chronic pain patients treated with spinal cord stimulation or dorsal root ganglia stimulation
Source: Health Qual Life Outcomes. 2023 Jul 20;21:77. doi: 10.1186/s12955-023-02158-2 (PMC10357671; doi:10.1186/s12955-023-02158-2)
Supplement: Supplementary file 1 — Additional file 1: Table Supplementary 1. Summary of all collected PROs (NRS, ODI, PCS, and PROMIS-29) and comparison of the mean of each PRO at all study visits and their corresponding significant differences. [file 12955_2023_2158_MOESM1_ESM.docx]

Supplement Table

Table S1.

|  | Follow Up | | | | | | | | | | | |
| --- | --- | --- | --- | --- | --- | --- | --- | --- | --- | --- | --- | --- |
|  | Baseline | | | Month 6 | | | Year 1 | | | Year 2 | | |
|  | Count | Mean | Standard Deviation | Count | Mean | Standard Deviation | Count | Mean | Standard Deviation | Count | Mean | Standard Deviation |
| NRS | N=701 | 7.5_a_ | (1.6) | N=452 | 4.1_b_ | (2.5) | N=305 | 4.2_b_ | (2.5) | N=115 | 4.3_b_ | (2.7) |
| ODI Total Score | N=701 | 49.8_a_ | (15.8) | N=452 | 35.3_b_ | (18.5) | N=305 | 35.8_b_ | (19.0) | N=115 | 35.3_b_ | (18.8) |
| PCS Total Score | N=701 | 26.5_a_ | (13.7) | N=452 | 16.1_b_ | (13.5) | N=305 | 15.1_b_ | (13.2) | N=115 | 12.6_b_ | (13.0) |
| PCS-Helplessness | N=701 | 11.9_a_ | (6.7) | N=452 | 7.1_b_ | (6.4) | N=305 | 6.6_b_ | (6.2) | N=115 | 5.5_b_ | (6.1) |
| PCS-Rumination | N=701 | 9.8_a_ | (4.6) | N=452 | 6.2_b_ | (5.1) | N=305 | 5.9_b_ | (4.8) | N=115 | 4.9_b_ | (4.9) |
| PCS-Magnification | N=701 | 4.8_a_ | (3.5) | N=452 | 2.8_b_ | (3.0) | N=305 | 2.6_b_ | (2.9) | N=115 | 2.3_b_ | (2.7) |
| PR-Pain Intensity | N=701 | 7.4_a_ | (1.6) | N=452 | 4.5_b_ | (2.4) | N=305 | 4.6_b_ | (2.5) | N=115 | 4.8_b_ | (2.5) |
| PR-Physical Function | N=701 | 34.5_a_ | (5.8) | N=452 | 38.6_b_ | (7.4) | N=305 | 38.3_b_ | (7.6) | N=115 | 37.9_b_ | (8.4) |
| PR-Anxiety | N=701 | 53.0_a_ | (17.0) | N=452 | 47.6_b_ | (17.6) | N=305 | 44.6_b_ | (17.8) | N=115 | 41.4_b_ | (18.0) |
| PR-Depression | N=701 | 54.2_a_ | (10.5) | N=452 | 52.2_a,b_ | (9.9) | N=305 | 50.9_b_ | (9.3) | N=115 | 49.5_b,c_ | (9.4) |
| PR-Fatigue | N=701 | 59.0_a_ | (9.1) | N=452 | 54.5_b_ | (9.6) | N=305 | 54.2_b_ | (10.2) | N=115 | 53.8_b_ | (10.7) |
| PR-Sleep Disturbance | N=701 | 57.7_a_ | (9.1) | N=452 | 53.6_b_ | (9.0) | N=305 | 52.8_b_ | (9.4) | N=115 | 52.0_b_ | (9.1) |
| PR-Social Roles | N=701 | 39.3_a_ | (8.0) | N=452 | 44.9_b_ | (8.9) | N=305 | 45.1_b_ | (9.3) | N=115 | 45.7_b_ | (10.1) |
| PR-Pain Interference | N=701 | 67.5_a_ | (5.9) | N=452 | 60.6_b_ | (8.1) | N=305 | 60.9_b_ | (8.4) | N=115 | 60.5_b_ | (8.8) |
| Note: Values in the same row and sub-table not sharing the same subscript are significantly different at p< .001 in the two-sided test of equality for column means. Cells with no subscript are not included in the test. Tests assume equal variances.^1^ | | | | | | | | | | | | |
| 1. Tests are adjusted for all pairwise comparisons within a row of each innermost sub-table using the Bonferroni correction. | | | | | | | | | | | | |
