# Supplementary figures and images for: Core patient-reported outcome measures for chronic pain patients treated with spinal cord stimulation or dorsal root ganglia stimulation
Source: Health Qual Life Outcomes. 2023 Jul 20;21:77. doi: 10.1186/s12955-023-02158-2 (PMC10357671; doi:10.1186/s12955-023-02158-2)

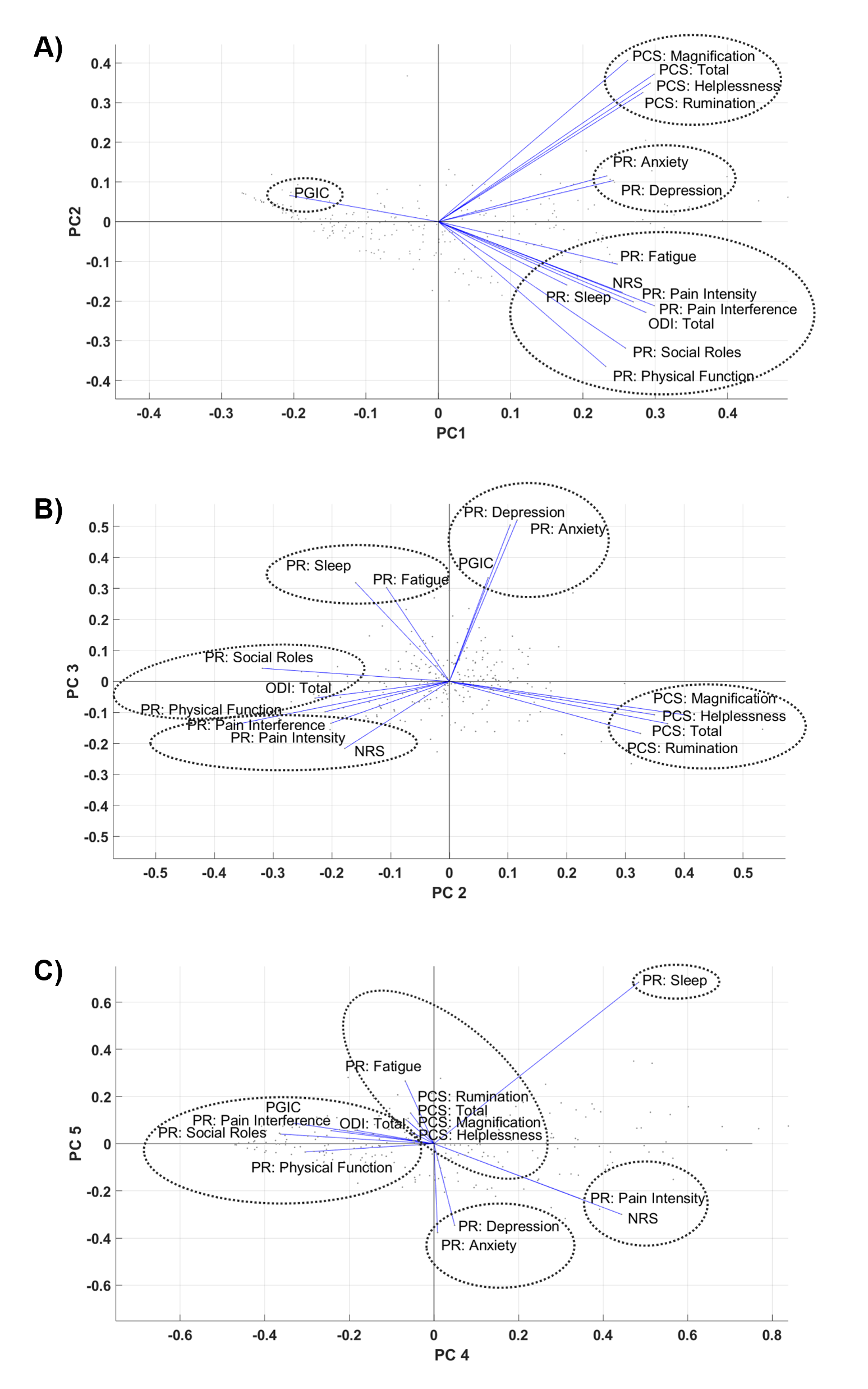

Supplement: Supplementary file 2 — Additional file 2: Figure Supplementary 1. Two-dimensional (2D) projection of the top 5 principal components used to cluster the questionnaires. Only ODI total score is included in the 2D projection of the principal components. [file 12955_2023_2158_MOESM2_ESM.bmp]

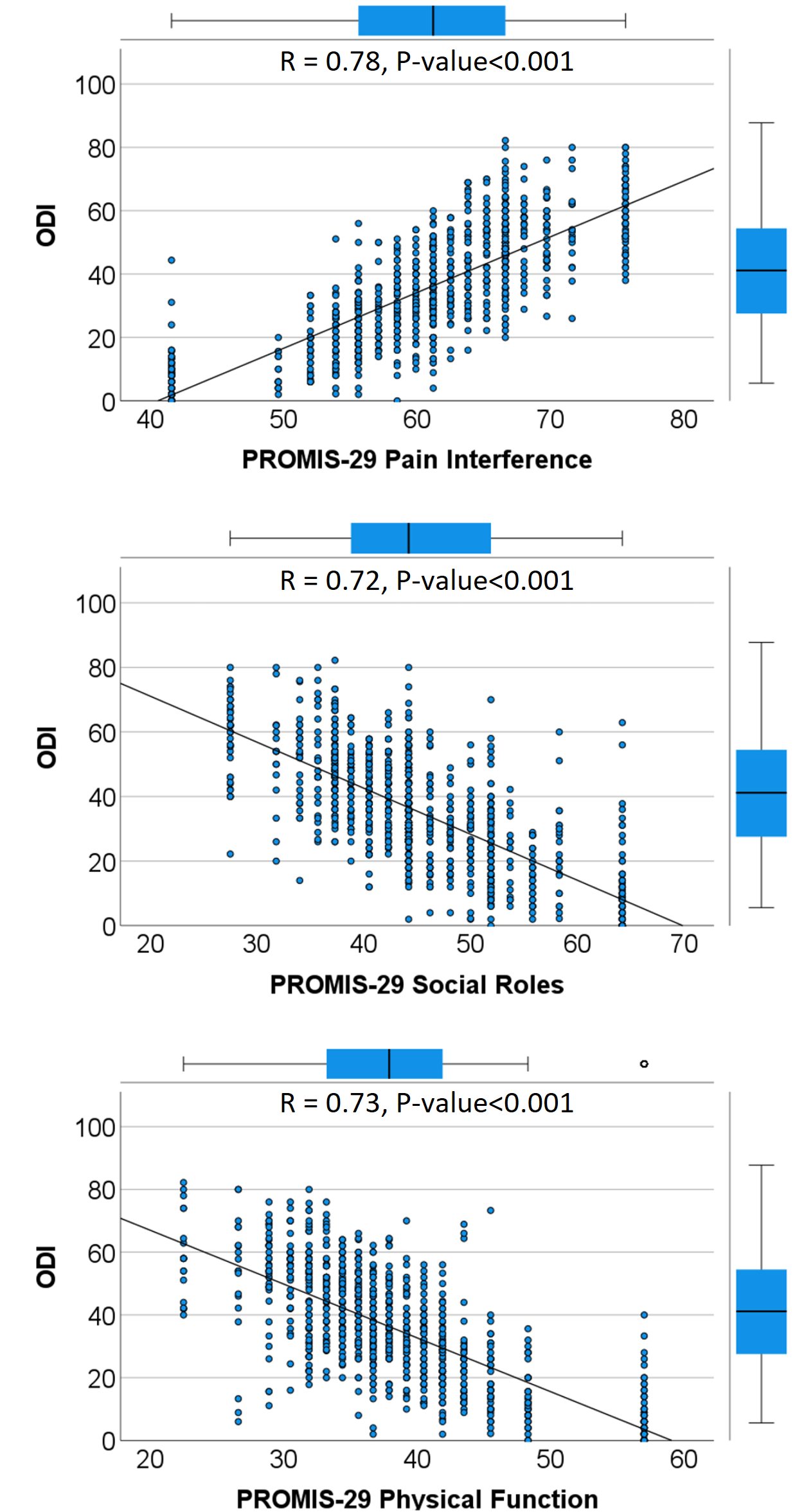

Supplement: Supplementary file 3 — Additional file 3: Figure Supplementary 2. Regression analysis of the ODI total score versus the PROMIS-29 domains of Physical Function, Social Roles, and Pain Interference. Each blue dot shows an individual subject data point for these two scales. The box and whisker plots show the variation of both scales; the minimum, the maximum, the first and third quartiles, and the median are illustrated with a solid black line. [file 12955_2023_2158_MOESM3_ESM.bmp]
